# Breaking Down Barriers … and Calcium: The EMPOWERment of Women With Calcific Coronary Artery Disease

**DOI:** 10.1016/j.jscai.2026.104383

**Published:** 2026-02-10

**Authors:** Michele L. Esposito, Suzanne J. Baron

**Affiliations:** a Division of Cardiology, Medical University of South Carolina, Charleston, South Carolina; b Division of Cardiology, Massachusetts General Hospital, Boston, Massachusetts

**Keywords:** coronary artery disease, clinical trials, intravascular lithotripsy

For decades, a significant data gap has plagued the field of interventional cardiology research. While coronary artery disease (CAD) remains one of the leading causes of mortality for women, the evidence base for the diagnosis and treatment of CAD is overwhelmingly derived from men. The management pathways for some aspects of CAD appear to perform equally as well for both sexes, but when it comes to the percutaneous treatment of calcified coronary lesions, which may not be the case. Compared with men, women with heavily calcified CAD are typically older, have smaller, more tortuous vessels and tend to present with a higher burden of comorbidities, all of which set them up for higher rates of intraprocedural complications and poorer long-term outcomes.^1^ There are multiple devices, including atherectomy and balloon-based tools, available to treat calcified CAD; however, data on their safety and efficacy in female patients have been sorely lacking, until now.

In this issue of *JSCAI*, McEntegart et al^2^ present the findings from the pivotal EMPOWER CAD (Equity in Modifying Plaque of Women With Undertreated Calcified Coronary Artery Disease) study.^2^ EMPOWER CAD was a prospective, multicenter, single arm, real-world observational study of women with de novo calcified CAD being treated with intravascular lithotripsy (IVL) as a first-line therapy. Exclusion criteria were minimal (eg, patients with cardiogenic shock, target lesion in-stent restenosis, or life expectancy <1 year), thereby allowing for a true all-comers population to be enrolled. The primary safety end point was target lesion failure (TLF) at 30 days, defined as a composite of cardiac death, myocardial infarction owing to the target vessel, or ischemia-driven target lesion revascularization. The primary efficacy end point was procedural success, defined as successful stent delivery with ≤30% residual stenosis without in-hospital TLF. In total, 399 women (448 lesions) were enrolled at 45 centers, and 448 lesions were treated with IVL. In this all-female cohort, IVL was found to be both efficacious (89.2% procedural success) and safe, with an 11.6% rate of TLF at 30 days. It is notable that the majority of TLF events (9.6%) were due to periprocedural myocardial infarction and that about half of these (52.6%) were due to asymptomatic cardiac enzyme elevation, as routine postprocedural biomarker collection was mandated in the protocol. Additionally, serious angiographic complications were exceedingly low with only 2.6% of patients noted to have type D or higher dissections after IVL treatment, all of which were resolved after stenting on final angiogram. Taken together, these findings convincingly demonstrate that IVL is a formidable tool in our armamentarium for the treatment of calcified CAD in women.

IVL has previously been shown to be safe and effective in the pivotal Disrupt CAD series of studies^3^, ^4^, ^5^; however, male patients comprised about 77% of this study population. In a sex-specific analysis of the pooled Disrupt CAD I, II, III, and IV studies, which allowed for the evaluation of 144 women, no significant sex-based differences in outcomes were noted, with women experiencing high rates of procedural success (91.7%) and low rates of serious angiographic complications (1.6%).^6^ These results are similar to what was observed in EMPOWER CAD, which studied a female population that was almost 3 times the size of all the Disrupt CAD studies, thereby demonstrating the procedural reproducibility of IVL performance in women.

Prior retrospective studies of atherectomy devices have shown higher rates of complications in women, such as dissection, cardiac tamponade, and bleeding,^7^ although some contemporary studies have not reproduced these findings.^8^ Although these studies are small (often <125 women), they still can induce hesitancy in the operator and thereby lead to the undertreatment of calcified lesions in women because of a desire to avoid complications. EMPOWER CAD serves to mitigate those concerns that we, as an interventional community, can feel comfortable performing IVL in our female patients and perhaps start to lessen sex-based disparities in the treatment of women with CAD.

In addition to promoting safe and effective calcium modification in women, EMPOWER CAD also provides a roadmap on how to close the gap regarding the underrepresentation of women in cardiac trials. While there are many barriers to enrolling women in clinical trials—including access, caregiving responsibilities, implicit provider bias, and restrictive eligibility criteria—lack of representative trial leadership likely also contributes. Indeed, although studies have demonstrated that enrolment of female patients in clinical trials is significantly higher when the principal investigator (PI) is a woman,^9^ a recent analysis evaluating 200 cardiology trial publications found that women comprised only 11.1% of trial leadership and that 41.5% had no female investigators involved at all.^10^ EMPOWER CAD broke that mold with both national PIs and 71% of site PIs being female. The results were not only the enrollment of a large women-only cohort of patients but that the trial also accomplished this feat months ahead of the projected study timeline.

In conclusion, EMPOWER CAD is a landmark study that truly sets the bar for trial implementation for studying women with cardiovascular disease. Certainly, it will be important to follow the study population over time (and 3-year follow up is planned) to ensure that the acute success of the IVL strategy translates into the longer term. Until then, we no longer need to rely on data extrapolated from male patients, who have different anatomic, physiologic, and clinical presentations. We finally have sex-specific evidence, demonstrating that women with calcified lesions can be treated safely and effectively. As we move toward a more inclusive era in interventional cardiology, we can feel empowered that we are indeed breaking through barriers and providing our female patients with optimal coronary revascularization.

## Peer review statement

Deputy Editor Suzanne J. Baron had no involvement in the peer review of this article and has no access to information regarding its peer review. Full responsibility for the editorial process for this article was delegated to Editor-in-Chief Alexandra J. Lansky.

## Declaration of competing interest

Suzanne J. Baron was a site principal investigator for EMPOWER CAD; receives institutional research support from Boston Scientific, Abiomed, and Acarix; and received consulting/ad board/speaking honoraria from Zoll Medical, Medtronic, Boston Scientific, Edwards Lifesciences, HeartFlow, Chiesi, and Pi-Cardia. Michele Esposito was a co-investigator for EMPOWER CAD.
